# The Relationship between Anthropometric Measurements and Vitamin D Levels and Insulin Resistance in Obese Children and Adolescents

**DOI:** 10.3390/children9121837

**Published:** 2022-11-27

**Authors:** Emrah Çığrı, Funda Çatan İnan

**Affiliations:** Faculty of Medicine, Kastamonu University, Kastamonu 37150, Turkey

**Keywords:** obesity, vitamin D, insulin resistance

## Abstract

Objective: Our investigation aimed to determine the effect of vitamin D levels on the development of insulin resistance in obese adolescents and children and the influences of anthropometric measurements on predicting the development of insulin resistance. Materials and Methods: In this study, demographic data, laboratory findings, and anthropometric measurements of 150 adolescents and children that had obesity diagnoses between May 2021 and September 2022 were evaluated retrospectively. Those with and without insulin resistance were studied with regard to vitamin D levels, biochemical parameters, and anthropometric measurements. Three groups of patients were created: those with low levels of vitamin D (<20 ng/mL), those with insufficient levels (20–30 ng/mL), and those having normal levels (≥30 ng/mL). Groups were compared in terms of homeostatic model score (HOMA-IR) and anthropometric measurements. Correlation analysis was carried out to ascertain the correlation of anthropometric measurements with HOMA-IR. To ascertain the cutoff, specificity, and sensitivity values of anthropometric parameters in predicting insulin resistance in patients, receiver operating characteristic (ROC) analysis was carried out. Results: Vitamin D levels of obese adolescents and children with insulin resistance were substantially lower than those without insulin resistance (*p* < 0.001). As the vitamin D level increased, all anthropometric measurements except for the body fat percentage decreased significantly with the HOMA-IR score (*p* < 0.05). HOMA-IR demonstrated a strong positive relation with waist circumference (r_s_ = 0.726, *p* < 0.001). Waist circumference had high specificity and sensitivity in predicting insulin resistance (87.3% and 87.4%, respectively). Conclusions: A significant relationship was observed between insulin resistance development and low levels of vitamin D in obese children and adolescents. As vitamin D levels increase, anthropometric measurements are more stable and do not increase. Waist circumference is the most effective anthropometric measurement for predicting the development of insulin resistance in obese adolescents and children.

## 1. Introduction

Poor adipose tissue, liver, and skeletal muscle response to endogenous insulin release is known as insulin resistance (IR). Cardiovascular pathologies including type 2 diabetes mellitus (DM), atherosclerosis, and hypertension (HT) play an important part in the development of several disease disorders, such as fatty liver disease, and polycystic ovary syndrome (PCOS) in females [1,2].

The clinical findings of insulin resistance vary according to the etiology and severity, and the mechanisms of formation of clinical findings are still unknown. In recent studies, it has been found that insulin resistance is higher in adolescents than in adults, and this is due to the β-cell destruction that takes place, especially in obese adolescents and children. In addition, significant β-cell destruction may happen prior to impairment of fasting glucose levels and glucose tolerance [3]. HT may develop in obese patients with IR, and this may originate from the partial atrial natriuretic peptide deficiency caused by IR [4].

The gold-standard method for insulin resistance diagnosis is the hyperinsulinemic–euglycemic clamp test. However, the fact that the test is difficult, time-consuming, and requires experience restricts its use. The homeostasis model (HOMA-IR), which is estimated using only fasting blood glucose and insulin measurements, is the most widely used diagnostic tool. A high HOMA-IR value means that IR is more severe [5].

Today, the HOMA-IR model is insufficient in determining IR. Therefore, this research aimed to analyze the effects of anthropometric parameters (body fat percentage (BFP), body fat mass (BFM), basal metabolic rate (BMR), body mass index (BMI)), waist circumference (WC), bodyweight for height (BWH)) of obese adolescents and children on predicting the IR presence and the relationship between vitamin D levels and IR development.

## 2. Materials and Methods

This retrospective research included 150 children and adolescents who were admitted to the Pediatric Outpatient Clinic with the complaint of abnormal weight gain for the first time between May 2021 and September 2022 and had a BMI ≥ 95 p for ≥ 2 years, and BWH percentage ≥ 97.7 p for <2 years, and a diagnosis of obesity. Patients with syndromic and genetic obesity; those with obesity due to endocrine dysfunction (such as Cushing’s syndrome or hypothyroidism); patients using sulfonylurea, steroids, tricyclic antidepressants, and antihypertensive drugs for a long time; and those whose HOMA-IR, vitamin D level, and anthropometric measurements (BMI, BWH, WC, BFM, BFP, and BMR) were not examined were not included in the research.

The patients were evaluated retrospectively by age, gender, anthropometric measurements, vitamin D level, uric acid level, and complete blood count parameters on the date that the first examination of the HOMA-IR score was recorded from the hospital information system.

BMI was calculated with the formula weight/height^2^ (kg/m^2^). By dividing body weight by ideal weight, BWH was computed. At the conclusion of expiration, the waist was measured circumferentially with the use of a tape measure starting from the middle between the iliac crest and the bottom border of the ribs. BFM, BFP, and BMR were measured with a Tanita MC-780S/ST brand pediatric segmental body analysis device. The HOMA-IR value was determined as (glucose) × (insulin)/405, and the limit for positivity was taken as >3.42 [6]. Vitamin D deficiency was present at values less than 20 ng/mL, and insufficient levels ranged from 20 to 30 ng/mL, whereas levels over 30 ng/mL were considered normal [7].

The participants were separated into two samples, namely, HOMA-IR (+) and HOMA-IR (–), and compared in terms of demographic data, anthropometric measurements, vitamin D levels, and other biochemical parameters. Additionally, anthropometric measurements and HOMA-IR scores were compared with three vitamin D level groups (deficient, insufficient, and normal).

Statistical Analysis: Pearson’s chi-square test was applied to determine the group comparison of categorical variables. The significance of differences between the means of two continuous variables was determined by the Mann-Whitney U-test for nonnormally distributed variables and the independent sample t-test for normally distributed variables. The significance of differences between the means of three continuous variables was determined by the Kruskal-Wallis H-test for non-normal distribution and the Mann-Whitney U-test for normal distribution. Spearman’s rank correlation analysis was conducted to analyze the correlation between HOMA-IR and BMI, BWH, WC, BFM, BFP, and BMR. Binary logistic regression was used to determine the effects of BMI, BWH, WC, and BFP among obese patients with HOMA-IR (+) and HOMA-IR (–). The threshold for the statistical significance was *p* < 0.05. All data analyses were conducted using SPSS26.00 (SPSS Inc., Chicago, IL, USA).

## 3. Results

**Figure 1:**
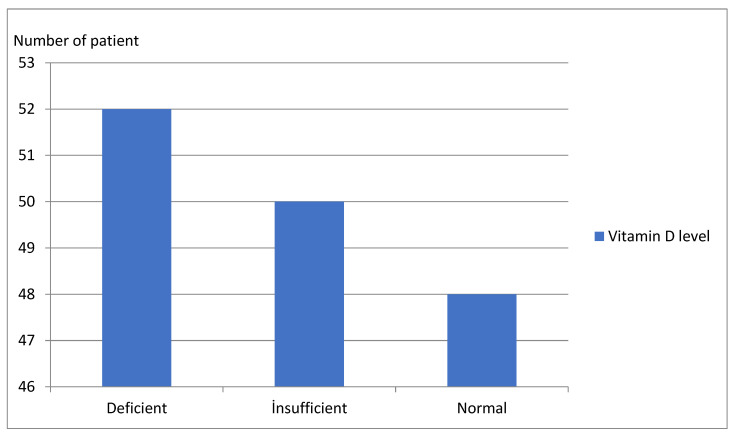
Number of patients by vitamin D levels.

Our study consisted of 150 obese children and adolescents. Of these, 78 (52%) were female, and 72 (48%) were male. Of the patients, 87 (58%) were HOMA-IR (+), and 63 (42%) were HOMA-IR (–). Table 1 shows the comparison of demographic data, vitamin D levels, and other biochemical parameters of the two groups. Vitamin D levels were significantly lower in HOMA-IR (+) obese patients than in HOMA-IR (–) obese patients (*p* < 0.001) (Figure 1). However, demographic information, uric acid levels, and complete blood count measures did not significantly differ between the two groups (*p* > 0.05) (Table 1).

Table 2 shows the comparison of the anthropometric measurements of the two groups. BMI, BWH, WC, BFM, BFP, and BMR were significantly higher in HOMA-IR (+) individuals than in HOMA-IR (–) individuals (*p* < 0.001) (Table 2).

When the patients were divided into three groups according to their vitamin D levels (deficiency, insufficiency, normal), there was a statistically significant difference between the three groups in terms of BMI (*p* = 0.003), BWH (*p* = 0.023), WC (*p* < 0.001), BFM (*p* = 0.004), BMR (*p* = 0.019), and HOMA-IR score (*p* < 0.001). In pairwise comparisons, these parameters were statistically significantly lower for patients with normal (25-OHD ≥ 30 ng/mL) levels of vitamin D than patients with deficient (25-OHD ≤ 20 ng/mL) levels of vitamin D (*p* < 0.05) (Table 3).

A correlation analysis of Spearman’s rank-order was conducted to understand the correlation between HOMA-IR and anthropometric measurements (Table 4). In the correlation table, a strong positive relation was present between HOMA-IR and WC (rs = 0.726, *p* < 0.001), and a moderate positive relation was present between BFM (rs = 0.467, *p* < 0.001) and BFP (rs = 0.431, *p* < 0.001) and HOMA-IR.

Dual regression models were developed to analyze the IR-related risk coefficients of anthropometric measurements of obese children and adolescents. Among the anthropometric measurements, WC had the greatest risk coefficients. Each unit increase in WC increased the risk of IR development by 4.394 times (Table 5).

**Figure 2:**
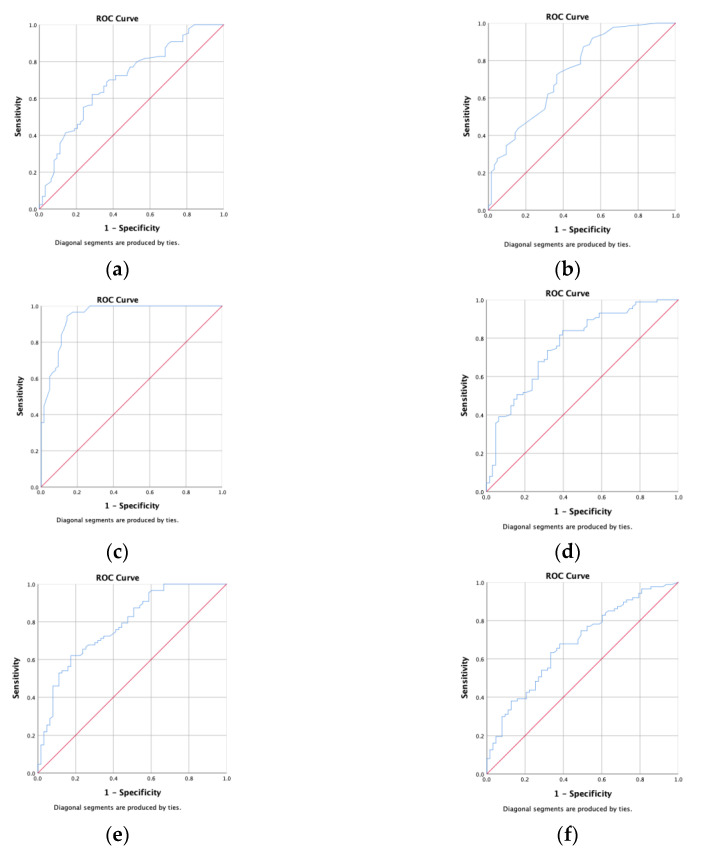
Receiver operating characteristic curves of the anthropometric indices. (**a**) BMI; (**b**) BWH; (**c**) WC; (**d**) BFM; (**e**) BFP (**f**) BMR.

The optimal cutoff, specificity, and sensitivity values of anthropometric measurement parameters were calculated by ROC analysis to predict the development of IR in obese adolescents and children. All were significant predictors of IR with comparable AUC values (*p* < 0.001) (Table 6, Figure 2).

The areas under the curve (AUCs) of BMI (Figure 2a), BWH (Figure 2b), WC (Figure 2c), BFM (Figure 2d), BFP (Figure 2e), and BMR (Figure 2f) were 0.701, 0.742, 0.945, 0.765, 0.782, and 0.682, respectively. ROC curves are in shown in Figure 1. The optimal cut-off values of BMI (Figure 2a), BWH (Figure 2b), WC (Figure 2c), BFM (Figure 2d), BFP (Figure 2e), and BMR (Figure 2f) were 24.15, 133.5, 69.0, 14.35, 297.95, and 1377.5, respectively. The WC with a cutoff point of 69 cm had the peak of the ROC curve with the highest capacity to exclude IR (sensitivity 87.4%, specificity 87.3%) (Figure 2c).

## 4. Discussion

This study investigated the variations in the anthropometric measurements of obese adolescents and children who developed insulin resistance and determined the relationship between levels of vitamin D and IR development. Obese children and adolescents with low vitamin D levels had a higher risk of developing IR. Similarly, anthropometric measurements increased significantly as vitamin D levels decreased. Among the anthropometric measurement parameters of obese children and adolescents, WC had the highest specificity and sensitivity in predicting IR development.

In parallel because of the rise in incidence of obesity in adolescents and children, the prevalence of IR also increased. In a study in the USA, the frequency of IR was 4.5% in children with normal weight, while this rate increased to 37.8% in obese children [8]. In a systematic study in 2015, the frequency of IR in children varied according to age groups, diagnostic criteria, and ethnicity, and this rate was 3.1–44.0% [9].

Through its endocrine effects, vitamin D is essential for glucose homeostasis and insulin secretion. Vitamin D is created in the skin by the action of sunshine (ultraviolet B). Deficiency of vitamin D, a pandemic problem in public health, is common in adolescents and obesity worldwide [10]. Vitamin D deficiency has an association with IR in adults, but the relationship between IR and vitamin D deficiency in obese children is still unclear [11]. Oda et al. [12] found that vitamin D deficiency disrupted the leptin/adiponectin ratio and caused IR development. Gün et al. [13] reported in their study on 92 obese children that there was a significant negative correlation between vitamin D level and serum insulin level and HOMA-IR and that vitamin D was associated with IR regardless of obesity. Lee et al. [10] found that vitamin D deficiency negatively affected IR and metabolic parameters. Delvin et al. [14] found that every 4 ng/mL increase in vitamin D levels decreased HOMA-IR. According to the study of Christine et al. [15], vitamin D deficiency was significantly associated with HOMA-IR alone in children, and there was a significant decrease in HOMA-IR with one-year vitamin D supplementation. Mengen et al. [16] found that obese children with IR had significantly lower vitamin D levels than those without IR, and those with low vitamin D levels had significantly more hepatosteatosis. Similarly, in other studies, a significant relationship was present between high HOMA-IR levels and vitamin D deficiency in obese children and adolescents [17,18,19]. On the other hand, Rajesh et al. [20] found that obese children with low vitamin D levels had higher blood sugar and insulin levels, but there was no significant relationship between IR and vitamin D levels. Similarly, in other studies, there was no significant relationship between vitamin D levels and insulin and HOMA-IR levels in obese children [21,22,23,24]. In this study, vitamin D levels were significantly lower in obese children and adolescents with IR than in those without IR. This study is in line with the majority of the literature, and the results indicate that low vitamin D levels in obese children and adolescents increase the risk of IR.

In obese individuals, vitamin D levels in the blood were lower due to the decrease in the production of vitamin D from the skin and intestinal absorption, the accumulation of vitamin D in the increased fat tissue, and the changing metabolism [25]. Hedyeh et al. [26] found a negative correlation between vitamin D levels and BMI in obese children. Similarly, Christine et al. [15] found a negative correlation between BMI and vitamin D levels. In the study of Rajesh et al. [20], there was a significant decrease in BFM and body fat mass index as the vitamin D level increased in obese children, but there was no significant difference in the WC and waist/hip ratio. In Lenders et al. [24], BFM and BFP were inversely correlated with vitamin D levels in obese adolescents. In our study, BMI, BWH, WC, and BFM were significantly inversely correlated with vitamin D levels. In light of this information, we can say that vitamin D is protective against the increase in body fat tissue in obese children and adolescents.

Adipose tissue acts as an endocrine organ and consists of visceral adipose tissue (VAT) and subcutaneous adipose tissue (SAT). These adipose tissues are associated with HOMA-IR by secreting free fatty acids into the bloodstream, and VAT is more effective on insulin variables than SAT [27]. VAT secretes more interleukin-6 (IL-6) and plasminogen activator inhibitor-1 (PAI-1) into the bloodstream. IL-6 and PAI-1 may be the main cause of increased IR in obese patients with increased VAT. If VAT is reduced by weight loss in obese patients, the secretion of IL-6 and PAI-1 decreases, resulting in significant improvement in insulin sensitivity [28].

The effect of the different adiposity factors that make up adipose tissue on IR is still unclear. BMI, BWH, WC, BFM, and BFP of obese children and adolescents with IR were significantly higher than those without IR. There was a strong positive correlation between HOMA-IR and WC and a moderate positive correlation between BFM and BFP and HOMA-IR. In the binary logistic regression analysis, the strongest parameter in predicting IR among anthropometric measurements was WC. Yang et al. [29] found that HOMA-IR was strongly positively correlated with WC and BFM and moderately positively correlated with BMI and BFP. Similar to our study, WC was a stronger anthropometric measurement than BMI to predict IR development. Similarly, Joseph et al. [30] reported that HOMA-IR was positively correlated with BFM and WC, and Rajesh et al. [20] reported that HOMA-IR was positively correlated with BFM. Marwa et al. [31] stated in their study that BMI and WC had significant AUCs in predicting IR in obese children and adolescents, and that BMI had the highest sensitivity and WC the highest specificity.

Recent cross-sectional studies have reported that obesity results in hyperuricemia, and that in addition to nephropathy and gout arthritis, hyperuricemia also contributes to IR, type 2 DM, and cardiovascular pathologies [32]. Increased VAT due to obesity produces uric acid (UA), and hyperuricemia leads to a significant increase in BMI and WC [33]. In the research of Yang et al. [34] on obese children and adolescents, the serum UA values of those with IR were significantly greater than those without IR, and there was a strong positive relation between HOMA-IR and UA. In this study, there was no significant difference between those with and without IR in terms of UA levels.

## 5. Conclusions

Low vitamin D levels in obese adolescents and children are a significant risk factor for IR development. Increased anthropometric measurements, especially WC, which are routinely examined in the follow-up of obese children, are a warning in terms of IR development. The main limitations of this study are that it is a retrospective study and includes only one center. The findings can be supported by prospective multi-center studies with larger sample sizes. Another limitation of our study is that we did not examine the patients according to age and BMI categories. The fact that the post-treatment processes of the patients were not included in our study is also a limitation of our study.

## Figures and Tables

**Table 1:** Sociodemographic and biochemical characteristics of the two samples.

Variables		Total *n* = 150	HOMA-IR (+) *n* = 87 [58%]	HOMA-IR (–) *n* = 63 [42%]	Test	*p*
Age(years)		8.0 [6.0–12.0]	8.0 [6.0–12.0]	8.0 [6.0–11.0]	2685.5 *	0.833
Gender	Female	78 [52%]	45 [57.6%]	33 [42.4%]	X2 = 0.006	0.937
	Male	72 [48%]	42 [58.3%]	30 [41.7%]		
Vitamin D (ng/mL) **		[20.18 ± 7.83]	[17.72 ± 7.32]	[23.57 ± 7.27]	−4.834	<0.001
Uric acid (mg/dL)		4.75 [4.10–5.50]	4.80 [4.00–5.70]	4.70 [4.10–5.30]	2606.5 *	0.610
MCV(fL)		[80.28 ± 4.37]	[80.68 ± 4.35]	[79.73 ± 4.36]	1.306	0.194
RDW (%)		13.15 [12.60–13.70]	13.20 [12.60–13.70]	13.00 [12.40–13.60]	2411.5 *	0.210
NLR		1.51 [1.18–1.98]	1.49 [1.15–1.86]	1.59 [1.19–2.01]	2618.0	0.641
PMI (fl/nL) **		[3231.33 ± 710.50]	[3397.23 ± 648.34]	[3140.32 ± 784.60]	1338	0.183

* Mann-Whitney U test (median of variables), chi-Square test (*n*; %), ** independent sample of *t*-test (mean ± SD), MCV: mean corpuscular volume, RDW: red cell distribution width, NLR: neutrophil/lymphocyte ratio, PMI: platelet mass index.

**Table 2:** Comparison of the anthropometric characteristics of the two groups.

Variables	Total *n* = 150	HOMA-IR (+) *n* = 87 (58%)	HOMA-IR (–) *n* = 63 (42%)	Test	*p*
BMI (kg/m^2^)	24.4 (21.97–27.47)	26.0 (22.7–28.4)	22.6 (20.9–25.6)	1641.5	<0.001
BWH (%)	135.0 (125.0–144.0)	138.0 (130.0–151.0)	127.0 (121.0–138.0)	1413.5	<0.001
WC (cm)	71.5 (55.0–89.0)	84.0 (72.0–95.0)	54.0 (50.0–60.0)	303.0	<0.001
BFM (kg)	15.05 (10.02–21.52)	18.0 (12.5–25.1)	10.8 (7.4–16.3)	1286.0	<0.001
BFP (%)	31.61 ± 8.44	35.11 ± 7.40	26.79 ± 7.38	6.801	<0.001
BMR (kcal)	1394.0 (1200.0–1622.25)	1445.0 (1277.0–1736.25)	1286.0 (1124.0–1488.0)	1750.0	<0.001

Mann-Whitney U test (median of variables), independent sample of *t*-test (mean ± SD).

**Table 3:** Comparison of HOMA-IR scores and anthropometric measurements of individuals with respect to vitamin D levels.

Vitamin D Level (ng/mL)	<20 *n* = 52 (34.6%)	20–30 *n* = 50 (33.4%)	≥30 *n* = 48 (32%)	Test	*p*
BMI (kg/m^2^)	27.95 (24.27–30.5)	25.9 (22.2–27.7)	23.0 (21.2–26.5)	1641.5	0.003
BWH (%)	141.0 (129.25–154.0)	135.0 (127.5–147.0)	134.0 (121.0–140.0)	1413.5	0.023
WC (cm)	87.5 (78.25–97.0)	80.0 (66.5–92.5)	60.0 (52.5–78.0)	303.0	<0.001
BFM (kg)	26.1 (10.77–36.2)	16.8 (11.5–22.2)	12.2 (7.75–19.2)	1286.0	0.004
BFP (%) *	36.29 ± 10.62	31.95 ± 8.02	30.47 ± 8.26	2.584	0.079
BMR (kcal)	1548.5 (1421.0–1633.0)	1408.0 (1237.5–1756.0)	1334.0 (1159.5–1483.0)	1750.0	0.019
HOMA-IR	6.8 (6.63–6.92)	4.98 (3.38–5.78)	1.99 (0.91–3.85)	0.000	<0.001

Kruskal-Wallis H test (median of variables), * one-way ANOVA (mean ± SD).

**Table 4:** Correlations between HOMA-IR score and anthropometric measurements.

	BMI	BWH	WC	BFM	BFP	BMR
r	0.387	0.398	0.726	0.467	0.431	0.347
*p*	<0.001	<0.001	<0.001	<0.001	<0.001	<0.001

**Table 5:** Dual logistic regression for prognostic factors in terms of insulin resistance development in obese adolescents and children.

Variables	β	SE	*p*	OR	95% CI for OR	
					Lower	Upper
Constant	33.002	10.443	0.002			
BMI	1.126	0.393	<0.001	1.374	2.033	9.498
BWH	1.229	0.080	0.004	1.555	0.680	0.930
WC	3.480	0.098	<0.001	4.394	0.539	0.791
BFP	1.280	0.108	0.010	1.795	0.611	0.934

**Table 6:** Area under the curve (AUC) and cutoff values of anthropometric indices to estimate insulin resistance between obese children and adolescents.

Variables	AUC	Asymptotic Sig.	Asymptotic 95% CI		Cutoff	Sensitivity (%)	Specificity (%)
			Lower	Upper			
BMI	0.701	<0.001	0.616	0.785	24.15	66.7	65.1
BWH	0.742	<0.001	0.662	0.822	133.5	66.7	65.1
WC	0.945	<0.001	0.908	0.982	69.0	87.4	87.3
BFM	0.765	<0.001	0.688	0.843	14.35	69.0	68.3
BFP	0.782	<0.001	0.708	0.856	297.95	69.0	68.3
BMR	0.681	<0.001	0.595	0.766	1377.5	64.4	63.5
